# Maternal near misses from two referral hospitals in Uganda: a prospective cohort study on incidence, determinants and prognostic factors

**DOI:** 10.1186/s12884-016-0811-5

**Published:** 2016-01-28

**Authors:** Annettee Nakimuli, Sarah Nakubulwa, Othman Kakaire, Michael O. Osinde, Scovia N. Mbalinda, Rose C. Nabirye, Nelson Kakande, Dan K. Kaye

**Affiliations:** Department of Obstetrics and Gynecology, School of Medicine, College of Health Sciences, Makerere University, P.O. Box 7072, Kampala, Uganda; Department of Obstetrics and Gynecology, Jinja Regional Hospital, Jinja, Uganda; Department of Nursing, School of Health Sciences, College of Health Sciences, Makerere University, P.O. Box 7072, Kampala, Uganda; Clinical, Operations and Health Services Research Program, Joint Clinical Research Centre, P. O. Box 10005, Kampala, Uganda

## Abstract

**Background:**

Maternal near misses occur more often than maternal deaths and could enable more comprehensive analysis of risk factors, short-term outcomes and prognostic factors of complications during pregnancy and childbirth. The study determined the incidence, determinants and prognostic factors of severe maternal outcomes (near miss or maternal death) in two referral hospitals in Uganda.

**Methods:**

A prospective cohort study was conducted between March 1, 2013 and February 28, 2014, where cases of severe pregnancy and childbirth complications were included. The clinical conditions included abortion-related complications, obstetric haemorrhage, hypertensive disorders, obstructed labour, infection and pregnancy-specific complications such as febrile illness, anemia and premature rupture of membranes. Near miss cases were defined according to the WHO criteria. Multivariate logistic regression analysis was conducted to identify prognostic factors for severe maternal outcomes.

**Results:**

Of 3100 women with severe obstetric complications, 130 (4.2 %) were maternal deaths and 695 (22.7 %) were near miss cases. Severe pre-eclampsia was the commonest morbidity (incidence ratio (IR) 7.0 %, case-fatality rate (CFR) 2.3 %), followed by postpartum haemorrhage (IR 6.7 %, CFR 7.2 %). Uterine rupture (IR 5.5 %) caused the highest CFR (17.9 %), followed by eclampsia (IR 0.4 %, CFR 17.8 %). The three groups (maternal deaths, near misses and non-life-threatening obstetric complications) differed significantly regarding gravidity and education level. The commonest diagnostic criteria for maternal near miss were admission to the high dependency unit (HDU) or to the intensive care unit (ICU). Thrombocytopenia, circulatory collapse, referral to a more specialized unit, intubation unrelated to anaesthesia, and cardiopulmonary resuscitation were predictive of maternal death (*p* < 0.05). Gravidity (ARR 1.4, 95 % C1 1.0–1.2); elevated serum lactate levels (ARR 4.5, 95 % CI 2.3–8.7); intubation for conditions unrelated to general anaesthesia (ARR 2.6 (95 % CI 1.2–5.7), cardiovascular collapse (ARR 4.9, 95 % CI 2.5–9.5); transfusion of 4 or more units of blood (ARR 1.9, 95 % CI 1.1–3.1); being an emergency referral (ARR 2.6, 95 % CI 1.2–5.6); and need for cardiopulmonary resuscitation (ARR 6.1, 95 % CI 3.2–11.7), were prognostic factors.

**Conclusions:**

The analysis of near misses is a useful tool in the investigation of severe maternal morbidity. The prognostic factors for maternal death, if instituted, might save many women with obstetric complications.

## Background

For every maternal death, there are about 100 women with severe maternal morbidity from life-threatening obstetric complications, referred to as maternal near misses [1]. A maternal near miss was defined by the World Health Organization (WHO) as “a woman who, being close to death, survives a complication that occurred during pregnancy, delivery or up to 42 days after the end of her pregnancy” [2]. Assessment of maternal near misses offers several advantages over assessment of maternal deaths: maternal near misses are commoner than maternal deaths. In addition, review of maternal near misses yields useful information on the pathways that lead to severe morbidity and death [3]. Furthermore, near miss assessment highlights the quality of obstetric care received by those who survive [3]. Prior to the WHO definition, the estimated incidence of maternal near misses varied in different studies, ranging from less than 1 per 1000 births to 82 per 1000 live births [4–11], partly as a result of different criteria that were used in the definition of maternal near miss. The World Health Organization (WHO) [2] developed a tool which utilize a combination of clinical signs/symptoms, management practices or presence of organ-system dysfunction. Maternal mortality represents the tip of an iceberg. For each death, many other women survive serious complications during pregnancy, delivery, or puerperium that lead to varying degrees of organ-system dysfunction [2]. In many cases, the causes of maternal death are also responsible for the cases of severe morbidity [2]. The WHO tool can therefore evaluate the quality of care provided to women presenting with severe morbidity.

Conceptually, maternal near misses represent a point on the continuum where good health and death are the extreme points [12]. On this continuum, women develop obstetric complications which could be described as uncomplicated, complicated (not life- threatening), severely complicated (life-threatening) or fatal [13–15]. Such individuals may recover, become temporarily or permanently disabled, or die [12]. Three approaches were proposed for definition of maternal near miss: using clinical features (signs, symptoms or clinical entities such as eclampsia or uterine rupture) [13]; using management practices (such as admission to intensive care) [11, 12], or using criteria of organ/system dysfunction [11]. The drawback of using the clinical criteria is the definition of the threshold of severity above which morbidity qualifies to be a maternal near miss. This threshold is context-specific as the probability of death depends on both the woman’s vulnerability to succumb and access to prompt quality care [1–5]. For instance, in studies from Benin [16], Uganda [6, 17], Angola [18, 19] and Burkina Faso [20], postpartum haemorrhage qualified as a maternal near miss only when additional events such as shock, blood transfusion or hysterectomy occurred. For studies that employed the management-based criteria, hysterectomy, admission to intensive care units [21, 22] and prolonged hospitalization [23–29] were the commonest procedures used [21, 22]. Indicators of severity of blood loss such as hypovolemia requiring massive blood transfusion, severe anaemia with hypotension (requiring intensive resuscitation) are used to identify maternal near miss. This criterion relies heavily on availability of management facilities.

The rationale of using the organ-system dysfunction-based criteria [11] is that women with such dysfunction are likely to die unless adequate prompt care is provided. For instance, obstetric haemorrhage constitutes a maternal near miss through vascular dysfunction (hypovolemia, shock and circulatory collapse), renal dysfunction (oliguria, acute kidney injury, renal failure), or coagulation dysfunction. The criteria rely heavily on availability of laboratory or other investigation facilities.

While assessment of maternal near miss is increasingly being recognised as potentially more useful that assessment of maternal mortality, severe maternal morbidity that constitutes a near miss is much less easy to define or quantify than maternal death. Studies that assessed maternal near miss using the WHO criteria recommended that future studies should evaluate the burden of maternal near miss using several morbidity and mortality indices [30, 31]. These indices include the *maternal near miss incidence ratios*, the *severe maternal outcome ratio*, the *case-fatality ratio, the severe maternal morbidity index*, and the *maternal mortality ratio.* To be able to reduce mortality in cases of severe maternal morbidity, there is a need to develop criteria for factors that can easily, uniformly and promptly identify prognostic factors among women with potentially life-threatening obstetric complications. There are few published studies from Africa that have used the WHO for definition of maternal near miss. Yet the WHO has recommended investigating near-misses as a benchmark practice for monitoring the quality of obstetric care and has standardized the criteria for diagnosis [2]. In addition, little is published on the burden of the maternal near miss as evidences by the morbidity severity indicators. The objective was to determine the incidence, characteristics, and prognostic factors for severe maternal outcomes (maternal near miss or maternal death) in two regional referral hospitals in central Uganda.

## Methods

### Study setting and design

This was a prospective cohort study of women admitted with pregnancy complications admitted between March 1, 2013 and February 28, 2014. The study was conducted at Mulago and Jinja Hospitals. Mulago is Uganda’s national referral hospital and the teaching hospital for Makerere University. The hospital has over 1500 beds, of which over 400 are maternity beds, and conducts over 30,000 deliveries per year. Jinja is a large regional referral hospital that serves about six district hospitals in Central and Eastern Uganda. It has a capacity of over 900 beds of which over 200 are maternity beds.

### Data collection

Participants were women with obstetric complications. In the selection criteria, all women with obstetric complications were enrolled into the study, irrespective of the severity of complications. Depending on the severity, the WHO criteria [2] were used to classify survivors of severe obstetric complications into maternal near miss morbidity (according to the criteria recommended by the tool), or those with non-life threatening obstetric complications. Women who consented to participate were recruited in the study. Using an interviewer-administered questionnaire, participant examination, investigations and through review of medical records, data was collected on socio-demographic characteristics, obstetric history, current pregnancy complications and pregnancy outcomes up to the time participants were discharged from hospital or died. Data for women with no or minor obstetric complications was excluded from the analysis.

### Sample size estimation

Assuming a power of 80 % at the 95 % significance level and a maximum accepted error of 5 %, and an assumed incidence ratio of obstetric complications of 15 % of all women who deliver, and assuming that 50 % of women with severe obstetric complications would end up as maternal near misses, our sample size was estimated to be 2600 women with obstetric complications of whom about 385 would be women with severe obstetric morbidity (near misses).

### Data analysis

We computed descriptive characteristics of maternal near miss, whereby categorical variables were presented as frequencies and percentages while numerical variables are presented as means or medians (with standard deviations or inter-quartile ranges respectively). In addition, we computed the following indicators: i) The *maternal near miss incidence ratios* derived as the ratio of near miss per 1000 live births respectively; ii.) The *severe maternal outcome ratio* as ratio of maternal death plus near misses per 1000 live births; iii) The *case-fatality rate for maternal complications* determined as the proportion of deaths out of the total number of patients presenting with specific complications, expressed as a percentage; iv) The *severe maternal mortality index*, derived as maternal deaths divided by (total deaths plus maternal near misses) expressed as a percentage; v) The *maternal mortality ratio* expressed as all maternal deaths per 100,000 live births as well as the perinatal mortality ratio expressed as all perinatal deaths per 1000 live births.

Furthermore, we analysed risk factors for severe maternal outcomes (maternal near miss or maternal death). Categorical variables were compared with *X*^*2*^ square or Fisher’s exact test and continuous variables with a two-tailed Student *t* test. We further analysed the prognostic factors of maternal near miss using log binomial regression analysis, where characteristics of near misses and maternal deaths were compared and adjusted relative risks computed. Variables included in the models were those with a *p*-value less than or equal to 0.2 or important from a clinical standpoint. After assessing the effects of confounding, interaction and collinearity, the final model contained mainly the laboratory-based and management-based criteria (for diagnosis of a maternal near miss).

### Ethical considerations

This research was part of a post-doctoral research project of the first author (DKK) entitled: *“Evaluation and surveillance of the impact of maternal and neonatal near-miss morbidity on the health of mothers and infants in Jinja and Mulago hospitals”.* Ethical approval to conduct the study was obtained from the Ethics and research committees of Mulago hospital (REC 310-2012), the School of Medicine, Makerere University College of Health Sciences (REC 2012-172) and from Uganda National Council for Science and Technology. Permission to conduct the study was obtained from the department of Obstetrics and Gynaecology, Makerere University, and from Mulago National Referral Hospital and Jinja Hospital.

Participants gave written informed consent to be enrolled in the study and for their data to be included in the study. Participants included minors (aged 14-17years), as Uganda national guidelines for human subject research [32] allow research on mature and emancipated minors in certain situations such as in pregnancy, with prior approval of an institutional review board. For those with very severe morbidity, consent was obtained retrospectively when they recovered, or consent was obtained from the next of kin to involve the patients’ in the study and to include the patients’ data in our dataset. Participants and their next of kin received assurances that participation was voluntary, and that participants were free to stop participation at any time without their decision affecting the care they were entitled to. All those with complications, and their newborns, were provided free medical care or, where necessary, were offered additional counselling or referred to get other support services not available at the two health facilities. Permission was obtained from the management of the two referral hospital (and from the study participants) to review the participants’ records.

## Results

Of the 3100 women with severe obstetric complications, there were 130 maternal deaths (4.2 %), 695 maternal near miss cases (22.7 %) and 2275 (73.4 %) women with non-life threatening obstetric complications (NLTC). In the same period, there were 25,840 live births. Table 1 shows the indicators for maternal and perinatal morbidity and mortality. The main cause was severe pre-eclampsia, with an incidence of 216 cases (7.0 %), but with a case fatality rate of only 2.3 %. Postpartum haemorrhage was the second main cause of morbidity, contributing to 208 of the 3100 cases (6.7 %) with a case-fatality rate of 7.2 %. However, uterine rupture caused the highest case-fatality of 27 out of 151 (17.9 %), followed by eclampsia (13 out of 171 or 17.8 %).

**Table 1:** Maternal perinatal and neonatal mortality indicators

Indicators	Ratio
Maternal near miss ratio	8.42 per 1000 live births.
Severe maternal outcome ratio	9.99 per 1000 live births
Maternal mortality ratio (MMR)	503 per 100,000 live births.
Severe maternal mortality index	15.8 %
Case-specific mortality rates	
Puerperal sepsis (9 out of 142)	6.3 %
Severe obstructed labor (19 out of 564)	3.4 %
*Abortion-related deaths*	
Abortion haemorrhage (5 out of 41)	12.2 %
Postabortion sepsis (5 of 114)	4.4 %
Overall (10 out of 155)	6.5 %
*Obstetric hemorrhage*	
Antepartum haemorrhage (5 out of 136)	5.1 %
Postpartum haemorrhage (15 out of 208)	7.2 %
Ruptured uterus (27 out of 151)	17.9 %
*Hypertensive disorders*	
Severe preeclampsia (5 out of 216)	2.3 %
Eclampsia (13 out of 171)	17.8 %
HELLP Syndrome (2 out of 7)	28.6 %
Overall (20 out of 394)	5.1 %

Table 2 shows the socio-demographic characteristics and medical history of the participants, stratified by severity of complications. The three groups differed significantly regarding their gravidity, education level and timing of the obstetric complications. Table 3 shows the obstetric complications displayed according to the maternal outcomes. In relation to the childbirth event, the most likely time for a mother to develop severe maternal outcomes was if they occurred in the intrapartum period and continued in the postpartum period (RR 2.5, 95 % CI 1.5–4.2, *p*-value <0.001); or if complications occurred in the postpartum period (RR 1.8, 95 % CI 1.0–3.0; *p*-value =0.044).

**Table 2:** Socio-demographic characteristics and medical history of the participants with obstetric complications displayed according to maternal outcomes

Characteristics	Number (percentage)	Number (percentage)	Number (percentage)	Number (percentage)	*p*-value (testing difference in groups)
	All patients	Maternal deaths	Maternal near miss	NLTC	
*Age category*					0.447
18 years or less	256 (8.3)	11 (8.5)	57 (8.2)	188 (8.3)	
19–24 years	1207 (38.9)	48 (36.9)	251 (36.1)	908 (39.9)	
> = 24 years	1637 (52.8)	71 (54.6)	387 (55.7)	1179 (51.7)	
*Gravidity*					<0.001
1	999 (32.2)	29 (22.3)	184 (26.5)	786 (34.6)	
2–4	1519 (49.0)	62 (47.7)	357 (51.4)	1100 (48.4)	
5 and more	582 (18.8)	39 (30.0)	154 (22.2)	389 (17.1)	
*Marital status*					0.249
Single	519 (16.7)	23 (17.7)	134 (19.3)	362 (15.9)	
Married	2571 (83.0)	107 (82.3)	560 (80.7)	1904 (83.9)	
Separated	10 (0.3)	0 (0.0)	0 (0.0)	10 (0.4)	
*Employment status*					0.053
Formal	304 (9.8)	9 (6.3)	68 (9.8)	228 (10.0)	
Informal	994 (32.1)	39 (30.2)	251 (36.2)	704 (31.0)	
Unemployed	1797 (58.1)	82 (63.6)	376 (56.0)	1343 (59.0)	
*ΩEducation level*					<0.001
None or primary level	1275 (41.4)	72 (56.3)	309 (44.7)	894 (39.5)	
Secondary level	1472 (47.8)	49 (38.2)	309 (44.7)	1114 (49.3)	
Post-secondary (tertiary)	333 (10.8)	7 (5.5)	75 (10.6)	253 (11.2)	
*Referral status*					0.121
Referred	2064 (66.7)	97 (74.6)	453 (65.3)	1514 (66.6)	
Not Referred (self-referrals)	1036 (33.3)	33 (25.4)	242 (34.7)	760 (33.4)	
*Timing of complications*					
Occurred before admission	1189 (53.8)	48 (36.9)	324 (46.6)	817 (35.9)	<0.001
Occurred before arrival and new complications developed	565 (25.6)	46 (27.6)	239 (34.4)	290 (12.8)	
Complications occurred during hospitalized	458 (20.6)	48 (35.4)	132 (18.9)	1168 (51.3)	
*Admission to the HDU*					
Yes	541 (17.5)	71 (54.6)	464 (66.8)	6 (0.3)	0.750
No	2559 (82.5)	59 (45.4)	231 (33.2)	2269 (99.7)	

*Key: NLTC* non-life-threatening obstetric complications

**Table 3:** Obstetric complications in women displayed according to the maternal outcomes

Characteristics	Number (percentage)	Number (percentage)	Number (percentage)	Number (percentage)	*p*-value
	All patients	Maternal deaths	Maternal near miss	NLTC	
	*N* = 3100	*N* = 130	*N* = 695	*N* = 2275	
*ΩObstetric Haemorrhage*					<0.001
Antepartum	136 (4.4)	7 (5.4)	82 (11.8)	47 (2.0)	
Postpartum	230 (7.4)	35 (26.9)	102 (14.7)	93 (4.0)	
Ruptured uterus	154 (5.0)	27 (20.8)	115 (16.5)	12 (0.5)	
*ΩAbortion-related*					0.007
Haemorrhage	41 (1.3)	5 (3.8)	23 (3.3)	13 (0.6)	
Postabortion sepsis	8 (0.3)	0 (0.0)	6 (0.9)	2 (0.1)	
Septic abortion	20 (0.6)	0 (0.0)	10 (1.4)	10 (0.4)	
*Hypertensive disorders*					<0.001
Severe Preeclampsia	218 (7.0)	5 (3.8)	79 (11.4)	134 (6.0)	
Eclampsia	172 (5.5)	13 (10)	132 (19.0)	27 (1.2)	
Chronic Hypertension	4 (0.1)	0 (0.0)	4 (0.6)	0 (0.0)	
HELLP Syndrome	9 (0.3)	2 (1.5)	7 (1.0)	0 (0.0)	
Puerperal sepsis	114 (3.7)	14 (10.8)	82 (11.8)	18 (0.8)	<0.001
Obstructed labor	564 (18.2)	19 (14.6)	42 (6.0)	503 (22.1)	<0.001
*Timing of the complications*					<0.001
&^a^Antepartum	1431 (48.1)	39 (30.0)	263 (37.8)	1129 (49.6)	
Postpartum	156 (5.2)	5 (3.9)	59 (8.5)	92 (4.0)	
Intrapartum	571 (19.2)	20 (15.6)	133 (19.1)	418 (18.4)	
Antepartum and intrapartum	449 (15.1)	34 (26.1)	91 (13.1)	324 (14.2)	
Intrapartum and postpartum	325 (10.9)	27 (20.0)	100 (14.4)	199 (8.8)	
Occurred in all three periods	45 (1.5)	6 (4.6)	49 (7.0)	113 (5.0)	
*∞Mode of delivery*					<0.001
Vaginal delivery	234 (15.5)	23 (28.4)	115 (21.2)	96 (10.8)	
βCaesarean section or laparotomy	1266 (83.8)	56 (69.1)	424 (78.1)	786 (88.7)	
Assisted delivery	10 (0.7)	2 (2.5)	4 (0.7)	4 (0.5)	

Ω Multiple responses, so percentage does not add up to 100 %; ^a^ Antepartum complications include sickle cell anemia, severe asthmatic attack and severe malaria in pregnancy; & All antenatal and abortion complications included in this group; ∞ Mode of delivery for 1510 women where delivery occurred; β Laparotomy for ruptured uterus

Table 4 shows the diagnostic criteria used for the definition of severe maternal outcomes (maternal near miss cases and maternal deaths) in the 192 women with obstetric complications. The commonest clinical criteria used to diagnose severe maternal outcomes were shock (as indicated by very low blood pressure or circulatory collapse and respiratory rate of more than 40 or less than 6 per minute). The commonest management-based criteria were admission to the HDU or ICU, and prolonged hospitalization longer than 7 days. The commonest laboratory-based criterion was thrombocytopenia (platelet count less than 100,000 per 100 ml). Shock, prolonged comatose state (for up to 12 h) and circulatory collapse pulse were predictive of maternal death (*p* < 0.05). Referral to a more specialized unit, admission to the HDU or ICU, intubation unrelated to anaesthesia, and cardiopulmonary resuscitation were management-based criteria that were predictive of a maternal death (*p* < 0.05). Laboratory-based diagnostic criteria were not predictive of maternal death (*p* > 0.05).

**Table 4:** Diagnostic criteria used for the definition of severe maternal outcomes (maternal near miss cases and maternal deaths) in the 3100 women with obstetric complications (including abortion-related complications)

Characteristics	All women	Maternal deaths	Maternal near miss	*p*-value
	Number *n* = 3100	Number (*n* = 130)	Number (*n* = 695)	
	N (%)	N (%)	N (%)	
*ΩClinical criteria*				
Cyanosis	442 (14.3)	69 (53.1)	370 (53.2)	0.973
Breathing rate more than 40 or less than 6 per minute	521 (16.8)	77 (59.2)	440 (63.3)	0.378
Oliguria poorly or unresponsive to fluids or diuretics	450 (14.8)	62 (47.7)	386 (55.7)	0.093
Loss of consciousness for up to 12 h	268 (8.7)	51 (39.2)	213 (30.6)	0.050
Unconscious (coma) with without recordable pulse	164 (5.3)	43 (33.1)	121 (17.4)	<0.001
Gasping due to low PaO2 or pulmonary edema	140 (4.5)	23 (17.7)	117 (17.8)	0.811
^a^Shock as indicated by very low blood pressure or circulatory collapse	606 (19.6)	72 (55.4)	533 (76.4)	<0.001
^a^Coagulation disorders evidenced by low platelets, elevated bleeding or clotting time, or bleeding tendency	362 (11.7)	50 (38.5)	310 (44.6)	0.195
Cerebrovascular accident	17 (0.6)	5 (3.8)	12 (1.7)	0.118
Paralysis	13 (0.4)	1 (0.8)	12 (1.6)	0.477
*ΩLaboratory-based criteria*				
^a^Bilirubin more than 100 mol/l or more than 6.0 mg/dL	274 (8.8)	44 (33.8)	226 (32.7)	0.792
^a^Thrombocytopenia (less than 100,000)	436 (14.1)	60 (46.2)	373 (53.7)	0.115
Creatinine more than 300 mol/l or more than 3.5 mg/dL	295 (9.5)	47 (36.2)	245 (35.3)	0.844
Elevated lactate	272 (8.8)	40 (30.8)	229 (33.0)	0.626
Low pH less than 7.1	255 (8.3)	31 (24.0)	221 (31.8)	0.071
*ΩManagement-based criteria*				
Oxygen saturation less than 90 % for more than 60 min	499 (16.1)	71 (54.6)	426 (61.3)	0.153
Use of vasoactive drug such as ephedrine	204 (6.6)	36 (27.7)	168 (24.2)	0.393
Dialysis for acute kidney failure	62 (2.0)	13 (10.0)	49 (7.2)	0.242
Peripartum hysterectomy cardiopulmonary	169 (5.5)	12 (9.2)	81 (11.7)	0.423
resuscitation	110 (3.6)	96 (73.8)	619 (88.9)	<0.001
^a^Transfusion more than 4 units of red blood cell concentrate	270 (8.7)	40 (30.8)	225 (32.4)	0.719
^a^Intubation unrelated to anesthesia	124 (4.1)	32 (24.6)	79 (11.4)	<0.001
Admission to the HDU or ICU	541 (17.5)	71 (54.6)	464 (66.8)	0.008
^a^Hospitalization longer than 7 days	537 (17.5)	34 (26.1)	496 (71.4)	<0.001
^a^Return to operation theatre	67 (2.2)	14 (10.8)	52 (7.4)	0.205
Referral to a more specialized unit	5 (1.5)	16 (14.0)	27 (3.9)	<0.001
Major operative non-obstetric surgery	9 (0.3)	2 (1.5)	7 (1.0)	0.592

Ω More than one criterion was manifest in some patients; *HDU* is the high dependency unit, *ICU* is Intensive care Unit; ^a^ Some patients were among those with NLTC

Table 5 shows the prognostic factors for maternal near miss. Gravidity (ARR 1.1, 95 % C1 1.0–1.2), elevated serum lactate levels (ARR 4.5, 95 % CI 2.3–8.7), intubation for conditions unrelated to general anaesthesia (ARR 2.6 (95 % CI 1.2–5.7), cardiovascular collapse(ARR 4.9, 95 % CI 2.5–9.5), transfusion of 4 or more units of blood (ARR 1.9, 95 % CI 1.1–3.1), being a referral (ARR 2.6, 95 % CI 1.2–5.6), and cardiopulmonary resuscitation (ARR 6.1, 95 % CI 3.2–11.7) were prognostic factors.

**Table 5:** Prognostic factors for severe maternal outcomes in women with severe maternal outcomes (maternal deaths plus near misses)

Characteristic	Maternal deaths	Maternal near miss morbidity	Crude risk ratios and 95 % CI	*p*-values	Adjusted risk ratios and 95 % CI	*p*-values
	N (%)	N (%)				
^a^ *Referral*						
Yes	16 (12.3)	27 (28.4)	3.5		2.6	
No	114 (87.7)	668 (71.6)	(1.8–6.6)	<0.001	(1.2–5.6)	0.013
*Admission to the ICU or HDU*						0.018
Yes	71 (54.6)	464 (66.8)	1.3	0.008	1.9	
No	59 (45.4)	231 (33.2)	(1.1–2.4)		(1.1–3.1)	
*CPR*						<0.001
Yes	34 (26.2)	76 (10.9)	2.9	<0.001	6.1	
No	96 (73.8)	619 (89.1)	(1.8–4.6)		(3.2–11.7)	
*Transfusion more than 4 units*						0.013
Yes	40 (30.8)	225 (32.3)	1.9		1.9	
No	90 (69.2)	470 (67.7)	(1.3–2.7)	0.002	(1.1–3.1)	
*Cardiovascular collapse*			2.9			<0.001
Yes	43(33.1)	121 (17.4)	(1.8–4.7)	<0.001	4.9	
No	87 (66.9)	574 (82.6)			(2.5–9.5)	
*Hypotension*						<0.001
Yes	72 (55.4)	531 (76.0)	2.6	<0.001	2.6	
No	58 (44.6)	164 (24.0)	(1.8–3.8)		(1.6–4.4)	
*Intubation*						<0.001
Yes	31 (31.3)	95 (13.7)	5.0		2.6	
No	99 (68.7)	600 (86.3)	(3.4–7.6)	<0.001	(1.2–5.7)	
*Elevated serum lactate*						<0.001
Yes	40 (30.8)	229 (32.9)	1.5	0.078	4.5	
No	90 (69.2)	466 (67.1)	(0.9–2.4)		(2.3–8.7)	
*βGravidity*						0.042
Gravid 1–4	100 (76.9)	541 (77.8)	1.2	0.046	1.1	
Gravida 5 or more	30 (23.1)	154 (22.2)	(1.1–1.4)		(1.0–1.2)	
*Thrombocytopenia*						0.337
Yes	60 (46.1)	373 (53.7)	1.4	0.116	1.4	
No	70 (53.9)	322 (46.3)	(0.9–1.9)		(0.7–3.20	

*HDU* high dependency unit, *ICU* intensive care unit, *RR* relative risk, *CI* confidence intervals, *CPR* cardiopulmonary resuscitation; ^a^ Referral to another unit outside the obstetric unit; β Comparing gravida 5 or more versus gravida 4 and less; ∞ 68 women died within the first 7 days after admission, and therefore not included in the analysis

## Discussion

The term near-miss describes a serious adverse event whereby death did not occur either due to luck or prompt adequate management [33]. This concept was defined by the World Health Organization (WHO) as “a woman who, being close to death, survives a complication that occurred during pregnancy, delivery or up to 42 days after the end of her pregnancy” [2]. The WHO criteria employ presence of organ dysfunction or a combination of clinical features, laboratory findings and management practices).

Our results show that the WHO maternal near miss tool [2] is useful in investigating both maternal morbidity and the quality of care provided to women with severe obstetric complications. This study is innovative in that it was conducted in two referral centres (the national referral and one regional referral hospital), the sample size is quite large, and the study design was prospective. In addition, the study used the WHO criteria for definition of maternal near-miss, and indicators of organ-system dysfunction were assessed as prognostic factors. In this study, the WHO tool enabled identification of nearly 7 times more cases of severe morbidity compared to what assessment of maternal mortality could have yielded. Severe pre-eclampsia was the commonest morbidity, followed by postpartum haemorrhage. However, uterine rupture caused the highest case-fatality followed by eclampsia. The commonest diagnostic criteria for maternal near miss were admission to the high dependency unit (HDU) or to the intensive care unit (ICU). Gravidity, elevated serum lactate levels, intubation for conditions unrelated to general anaesthesia, cardiovascular collapse, transfusion of 4 or more units of blood, being a referral, and need for cardiopulmonary resuscitation were prognostic factors.

Our finding show no differences on socio-demographic characteristics (except for education level and gravidity) and medical history between the three groups (NLTC, near miss cases and maternal deaths). Therefore, the different clinical causes of morbidity differ only according to severity of the complications. In addition, the outcomes may not depend so much on the socio-demographic or medical history as on the quality of care individuals receive. The outcome of critically ill patients, such as patients with severe obstetric complications, is dependent on clinical and individual factors, previous health status, physiologic reserve, disease severity and adequacy of care provided [34, 35]. The severity depends on the inherent risk of disease progression and the quality of care received in terms of timeliness, adequacy and comprehensiveness. Since near miss cases share characteristics with maternal deaths, they may be used to provide information about hurdles that needed to be overcome after onset of or worsening of complications. In that way, near misses provide invaluable information on obstetrical care.

The WHO maternal near miss tool may be used as a scoring tool for severe obstetric morbidity. The complications that are unique to pregnancy or childbirth and the changed physiologic parameters (as a result of pregnancy changes) make pregnancy, childbirth and the puerperium unique situations where routinely used scoring systems for disease severity may be inappropriate or inadequate [35, 36]. Many of the available scoring systems of disease are not applicable to disease severity in obstetric complications [34, 35]. Indeed the traditional risk stratification models used in critically ill non-obstetric patients usually overestimate mortality among pregnant women, which makes analysis of morbidity data especially prognostic factors and their interpretation difficult [36, 37].

The WHO maternal near miss tool is an innovative concept that could be used to identify prognostic indicators for patients with severe maternal morbidity [37, 38]. Early recognition and prompt management of severe life-threatening maternal morbidity through improved access, availability and affordability of critical life-saving skills and therapeutics is key to reduction of maternal morbidity and mortality. This calls for tools to assess prognostic factors in maternal near miss cases.

The Sequential Organ Failure Assessment (SOFA) score is a validated score used to quantify organ dysfunction and predict prognosis for severely ill persons admitted to the ICU [39, 40]. The SOFA score is one of the scoring systems used to track a patient’s status during the stay in an ICU where it is used to determine the extent of a person’s organ function or rate of failure. The score is based on six different scores, one each for the respiratory, cardiovascular, hepatic, coagulation, renal and neurological systems. Both the mean and highest SOFA scores are predictive of mortality outcomes: an increase in SOFA score during the first 24 to 48 h in the ICU predicts a mortality rate in 50–95 % of cases [39, 40]. In a study of obstetric patients admitted to ICU, which compared scores on the WHO tool and the total maximum Sequential Organ Failure (SOFA) score as the gold standard, the WHO near miss criteria had a sensitivity and specificity of 99.2 % and 86.0 % respectively for identification of organ failure in at least one organ system [39]. In addition, the WHO tool had a sensitivity of 100 % and specificity 70.4 % for prediction of maternal deaths [39]. The total maximum SOFA score had a good performance (area under the curve of 0.897) for prediction of cases of maternal near miss according to the WHO criteria [39]. In another study assessing the utility of the SOFA score in obstetric patients, the total maximum SOFA score was significantly higher in women with severe maternal morbidity (SMM) when compared to that in women without SMM (*p* < 0.001) [40]. In addition, the total maximum SOFA score was predictive of survival by being able to discriminate pregnant women with SMM who did not survive (AUROC 0.77, 95 % CI: 0.46, 1.00) [40]. The WHO tool is therefore useful in identification of organ-system dysfunction and multiple organ failure, which is the final cause of death in patients with severe obstetric complications.

The analysis of maternal near misses is a useful innovation in the investigation of severe maternal morbidity [41], though it may require modification in certain contexts. For instance, in a study conducted in Tanzania using a modification of the tool [42], 216 maternal near misses and 32 maternal deaths were identified over a two year-period. From a hospital based study that used a modification of the WHO tool [43], the maternal mortality ratio was 350 maternal deaths per 100,000 live births, the maternal near miss incidence ratio was 23.6 per 1000 live births, and the overall case fatality rate was 12.9 %. The use of the WHO tool underscores the practical challenges in determining organ-system dysfunction in obstetric patients. The evidence that the WHO tool scores fairly well in recognising organ dysfunction and failure when compared with standard tools such as the SOFA for assessing organ failure [44, 45], increases its utility in obstetric patients.

## Conclusion

In conclusion, the WHO tool for analysis of maternal near miss, which uses defined criteria, can identify more preventable causes of maternal death than the traditional clinical criteria alone. Prospective monitoring of maternal morbidity may be useful in identifying determinants and prognostic factors of severe maternal morbidity.

## Abbreviations

ARR: adjusted risk ratio
CI: confidence intervals
CPR: cardiopulmonary resuscitation
HDU: high dependency obstetric unit
HELLP syndrome: syndrome of hemolysis, elevated liver enzymes and low platelets
ICU: intensive care unit
NLTC: non-life threatening obstetric complication
RR: risk ratio
SIDA: Swedish International Development Agency
SMM: severe maternal morbidity
SOFA: sequential organ failure assessment
WHO: World Health Organization

## References

[1] Report on the World Health Organization working group on the classification of maternal deaths and severe maternal morbidities. Geneva: World Health Organization; 2009.

[2] Say L, Souza JP, Pattinson RC (2009). Maternal near miss – towards a standard tool for monitoring quality of maternal health care. Best Pract Res Clin Obstet Gynecol.

[3] Ronsmans C, Filippi V (2004). Beyond the numbers Reviewing maternal deaths and complications to make pregnancy safer. Reviewing severe maternal morbidity and learning from women who survive life threatening complications.

[4] Adisasmita A, Deviany PE, Nandiaty F, Stanton C, Ronsmans C (2008). Obstetric near-miss and deaths in public and private hospitals in Indonesia. BMC Pregnancy Childbirth.

[5] Souza JP, Cecatti JG, Parpinelli MA, de Sousa MH, Serruya SJ (2006). Systematic review of near miss maternal morbidity. Cad Saude Publica.

[6] Minkauskien M, Nadisauskiene R, Padaiga Z, Makari S (2004). Systematic review on the incidence and prevalence of severe maternal morbidity. Medicina.

[7] Okong P, Byamugisha J, Mirembe F, Byaruhanga R, Bergstrom S (2006). Audit of severe maternal morbidity in Uganda-implications for quality of care. Acta Obstet Gynecol Scand.

[8] Souza JP, Cecatti JG, Parpinelli MA, Serruya SJ, Amaral E (2007). Appropriate criteria for identification of near-miss maternal morbidity in tertiary care facilities: a cross sectional study. BMC Pregnancy Childbirth.

[9] Gandhi MN, Welz T, Ronsmans C (2004). An audit of life-threatening maternal morbidity in rural South Africa using ‘near-miss’ criteria adapted for primary level hospitals. Int J Gynecol Obstet.

[10] Waterstone M, Bewley S, Wolfe C (2001). Incidence and predictors of severe obstetric morbidity: case-control study. BMJ.

[11] Mantel GD, Buchmann E, Rees H, Pattinson RC (1998). Severe acute maternal morbidity: a pilot study of a definition for a near-miss. Br J Obstet Gynecol.

[12] Geller SE, Rosenberg D, Cox SM, Brown ML, Simonson L, Driscoll CA (2004). The continuum of maternal morbidity and mortality: factors associated with severity. Am J Obstet Gynecol.

[13] Stones W, Lim W, Al-Azzawi F, Kelly M (1991). An investigation of maternal morbidity with identification of life threatening ‘near miss’ episodes. Health Trends.

[14] Glazener CM, Abdalla M, Stroud P, Naji S, Templeton A, Russell IT (1995). Postnatal maternal morbidity: extent, causes, prevention and treatment. Br J Obstet Gynaecol.

[15] Waterstone M, Wolfe C, Hooper R, Bewley S (2003). Postnatal morbidity after childbirth and severe obstetric morbidity. BJOG.

[16] Filippi V, Alihonou E, Mukantaganda S, Graham WJ, Ronsmans C (1998). Near misses: maternal morbidity and mortality. Lancet.

[17] Kaye D, Mirembe F, Aziga F, Namulema B (2003). Maternal mortality and associated near-misses among emergency intrapartum obstetric referrals in Mulago Hospital, Kampala, Uganda. East Afr Med J.

[18] Strand RT, Campos PA, Paulsson G, de Oliveira J, Bergström S (2009). Audit of referral of obstetric emergencies in Angola: a tool for assessing quality care. Afr J Reprod Health.

[19] Strand RT, Tumba P, Niekowal J, Bergström S (2010). Audit of cases with uterine rupture: a process indicator of quality of obstetric care in Angola. Afr J Reprod Health.

[20] Filippi V, Ronsmans C, Gohou V, Goufodji S, Lardi M, Sahel A (2005). Maternity wards or emergency obstetric rooms? Incidence of near-miss events in African hospitals. Acta Obstet Gynecol Scand.

[21] Baskett TF, Sternadel J (1998). Maternal intensive care and near-miss mortality in obstetrics. Br J Obstet Gynecol.

[22] Pattinson RC, Say L, Makin JD, Bastos MH (2005). Critical incident audit and feedback to improve perinatal and maternal mortality and morbidity. Cochrane Database Syst Rev.

[23] Amata AO (1998). Anaesthetic and intensive care management of rupture of the gravid uterus: a review of 50 cases. Trop Doct.

[24] Fawzi HW, Kamil KK, Stronge J (1998). Rupture of the uterus in labour: a review of 14 cases in general hospital. J Obstet Gynecol.

[25] Oladapo OT, Ariba AJ, Odusoga OL (2007). Changing patterns of emergency obstetric care at a Nigerian University hospital. Int J Gynecol Obstet.

[26] Oladapo OT, Sule-Odu AO, Olatunji OA, Daniel OJ (2005). “Near-miss” obstetric events and maternal deaths in Sagamu, Nigeria: a retrospective study. Reprod Health.

[27] Prual A, Bouvier-Colle MH, de Bernis L, Breart G (2000). Severe maternal morbidity from obstetric causes in West Africa: incidence and case fatality rates. Bull WHO.

[28] Prual A, Huguet D, Garbin O, Rabe G (1998). Severe obstetric morbidity of the third trimester, delivery and early puerperium in Niamey (Niger). Afr J Reprod Health.

[29] Kaye DK, Kakaire O, Osinde MO (2011). Maternal morbidity and near-miss mortality among women referred for emergency obstetric care in rural Uganda. Int J Gynaecol Obstet.

[30] Haddad SM, Cecatti JG, Souza JP, Sousa MH, Parpinelli MA, Costa ML (2014). Applying the maternal near miss approach for the evaluation of quality of obstetric care: a worked example from a multicenter surveillance study. Biomed Res Int.

[31] Souza JP, Cecatti JG, Haddad SM, Parpinelli MA, Costa ML, Katz L (2012). The WHO maternal near-miss approach and the maternal severity index model (MSI): tools for assessing the management of severe maternal morbidity. PLoS One.

[32] Uganda National Council for Science and Technology (UNCST). National guidelines for research involving humans as research participants; July 2014. Kampala: UNCST. p.16-22 http://www.uncst.go.ug/dmdocuments/Human%20Subjects%20Protection%20Guidelines%20July%202014.pdf

[33] Nashef SA (2003). What is a near miss?. Lancet.

[34] Strand K, Flaatten H (2008). Severity scoring in the ICU: a review. Acta Anaesthesiol Scand.

[35] Higgins TL (2007). Quantifying risk and benchmarking performance in the adult intensive care unit. J Intensive Care Med.

[36] Lapinsky SE, Hallett D, Collop N, Drover J, Lavercombe P, Leeman M (2011). Evaluation of standard and modified severity of illness scores in the obstetric patient. J Crit Care.

[37] Lotufo FA, Parpinelli MA, Haddad SM, Surita FG, Cecatti JG (2012). Applying the new concept of maternal near-miss in an intensive care unit. Clinics.

[38] Cecatti JG, Souza JP, Parpinelli MA, Haddad SM, Camargo RS, Pacagnella RC (2009). Brazilian network for the surveillance of maternal potentially life threatening morbidity and maternal near-miss and a multidimensional evaluation of their long term consequences. Reprod Health.

[39] Cecatti JG, Souza JP, Oliveira Neto AF, Parpinelli MA, Sousa MH, Say L (2011). Pre-validation of the WHO organ dysfunction based criteria for identification of maternal near miss. Reprod Health.

[40] Kallur DS, Bada VP, Reddy P, Pandya S, Nirmalan PK (2014). Dysfunction and organ failure as predictors of outcomes of severe maternal morbidity in an obstetric intensive care unit. J Clin Diagn Res.

[41] Jayaratnam S, De Costa C, Howat P (2011). Developing an assessment tool for maternal morbidity near miss – a prospective study in a large Australian regional hospital. ANZJOG.

[42] Nelissen E, Mduma E, Broerse J, Ersdal H, Evjen-Olsen B, van Roosmalen J (2013). Applicability of the WHO maternal near miss criteria in a low-resource setting. PLoS One.

[43] Nelissen EJ, Mduma E, Ersdal HL, Evjen-Olsen B, van Roosmalen JJ, Stekelenburg J (2013). Maternal near miss and mortality in a rural referral hospital in northern Tanzania: a cross-sectional study. BMC Pregnancy Childbirth.

[44] Moreno R, Vincent JL, Matos R, Mendonça A, Cantraine F, Thijs L (1999). The use of maximum SOFA score to quantify organ dysfunction/failure in intensive care. Results of a prospective, multicenter study. Intensive Care Med.

[45] Vincent J-L, De Mendonça A, Cantraine F, Moreno R, Takala J, Suter PM (1998). Use of the SOFA score to assess the incidence of organ dysfunction/failure in intensive care units: results of a multicentric, prospective study. Working group on “sepsis-related problems” of the European Society of Intensive Care Medicine. Crit Care Med.

